# Phase 2 study of axicabtagene ciloleucel in Japanese patients with relapsed or refractory large B-cell lymphoma

**DOI:** 10.1007/s10147-021-02033-4

**Published:** 2021-10-01

**Authors:** Koji Kato, Shinichi Makita, Hideki Goto, Junya Kanda, Nobuharu Fujii, Kazuyuki Shimada, Koichi Akashi, Koji Izutsu, Takanori Teshima, Natsuko Fukuda, Tokuhito Sumitani, Hiroyuki Sumi, Shinji Shimizu, Yasuyuki Kakurai, Kenji Yoshikawa, Kensei Tobinai, Noriko Usui, Kiyohiko Hatake

**Affiliations:** 1 Department of Medicine and Biosystemic Science, Kyushu University Graduate School of Medical Sciences, 3-1-1, Maidashi, Fukuoka Higashi-ku, Fukuoka, 812-8582 Japan; 2 Department of Hematology, National Cancer Center Hospital, 5-1-1, Tsukiji , Chuo, Tokyo 104-0045 Japan; 3 Department of Hematology, Hokkaido University Faculty of Medicine, Kita 15, Nishi 7, Kita-ku, Sapporo, 060-8638 Japan; 4 Department of Hematology and Oncology, Graduate School of Medicine, Kyoto University, Yoshida-Konoe-cho, Sakyo-ku, Kyoto, 606-8501 Japan; 5 Department of Hematology and Oncology, Okayama University Hospital, 2-5-1 Shikata-cho, Kitaku, Okayama 700-8558 Japan; 6 Department of Hematology and Oncology, Nagoya University Graduate School of Medicine, 65 Tsurumai-cho, Showa-ku, Nagoya, 466-8550 Japan; 7 Daiichi Sankyo Co., Ltd, 1-2-58, Hiromachi, Shinagawa, Tokyo 140-8710 Japan; 8 Geriatric Health Services Facility Rehabilitation Care Funabashi, 4-8-30 Honcho, Funabashi, Chiba 273-005 Japan; 9 Department of Clinical Oncology and Hematology, The Jikei University Daisan Hospital, 4-11-1 Izumihoncho, Komae, Tokyo 201-8601 Japan; 10 Department of Lymphoma/Hematology Center, Mita Hospital, International University of Health and Welfare, 1-4-3 Mita, Minato, Tokyo 108-8329 Japan

**Keywords:** Axicabtagene ciloleucel, CD19-specific chimeric antigen receptor, Japan, KTE-C19, Non-Hodgkin lymphoma

## Abstract

**Background:**

Axicabtagene ciloleucel (axi-cel) is an autologous chimeric antigen receptor T-cell based anti-CD19 therapy. The ZUMA-1 study, multicenter, single-arm, registrational Phase 1/2 study of axi-cel demonstrated high objective response rate in patients with relapsed/refractory large B-cell lymphoma. Here, we present the results of the bridging study to evaluate the efficacy and safety of axi-cel in Japanese patients (JapicCTI-183914).

**Methods:**

This study was the phase 2, multicenter, open-label, single-arm trial. Following leukapheresis, axi-cel manufacturing and lymphodepleting chemotherapy, patients received a single infusion of axi-cel (2.0 × 10^6^ cells/kg). Bridging therapy between leukapheresis and conditioning chemotherapy was not allowed. The primary endpoint was objective response rate.

**Results:**

Among 17 enrolled patients, 16 received axi-cel infusion. In the 15 efficacy evaluable patients, objective response rate was 86.7% (95% confidence interval: 59.5–98.3%); complete response/partial response were observed in 4 (26.7%)/9 (60.0%) patients, respectively. No dose-limiting toxicities were observed. Grade ≥ 3 treatment-emergent adverse events occurred in 16 (100%) patients—most commonly neutropenia (81.3%), lymphopenia (81.3%) and thrombocytopenia (62.5%). Cytokine release syndrome occurred in 13 (81.3%) patients (12 cases of grade 1 or 2 and 1 case of grade 4). No neurologic events occurred. Two patients died due to disease progression, but no treatment-related death was observed by the data-cutoff date (October 23, 2019).

**Conclusion:**

The efficacy and safety of axi-cel was confirmed in Japanese patients with relapsed/refractory large B-cell lymphoma who have otherwise limited treatment options.

**Trial registration:**

JapicCTI-183914.

**Supplementary Information:**

The online version contains supplementary material available at 10.1007/s10147-021-02033-4.

## Introduction

Non-Hodgkin lymphoma (NHL) is the tenth most common type of cancer in Japan and diffuse large B-cell lymphoma (DLBCL) is the most common subtype [1, 2]. Although the combination of rituximab, cyclophosphamide, doxorubicin, vincristine, and prednisone (R-CHOP) has improved the prognosis of DLBCL patients, 30–50% of them are not cured by this treatment [3]. SCHOLAR-1, a pooled retrospective analysis of patients with refractory DLBCL showed a median overall survival (OS) of 6.3 months and an objective response rate (ORR) of 26% [4]. Axicabtagene ciloleucel (axi-cel, KTE-C19) is one of the autologous chimeric antigen receptor (CAR) T-cell therapies targeting CD19 which is considered as an optimal therapeutic target due to its uniform expression on malignant B-cells [5]. While axi-cel employs CD28 as a costimulatory domain [6], other anti-CD19 CAR T cells such as tisagenlecleucel (tisa-cel) [7] and lisocabtagene maraleucel (liso-cel) [8] employ 4-1BB as a costimulatory domain. ZUMA-1 (NCT02348216), multicenter, international (not including Japan), pivotal phase 1/2, single-arm study of axi-cel, demonstrated 82% of ORR, 54% of complete response (CR) rate, and 25.8 months of median OS in the relapsed/refractory (R/R) large B-cell lymphoma patients [6, 9–11].

To confirm the efficacy and safety of axi-cel in Japanese patients, J201 bridging study was conducted (JapicCTI-183914). In this paper, results of this bridging study are reported.

## Patients and methods

### Study design

This was a phase 2, multicenter, open-label, single-arm study to evaluate the efficacy and safety of axi-cel in Japanese patients with R/R large B-cell lymphoma.

**Fig. 1:**
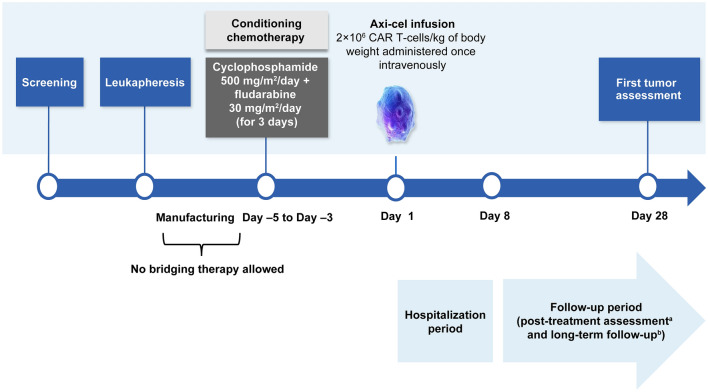
Study design. ^a^Week 2, week 4, month 2, and month 3. ^b^Every 3 months ± 2 weeks to month 18, every 6 ± 1 months from month 24 to month 60, and visit once a year ± 3 months from years 6–15. *axi-cel* axicabtagene ciloleucel, *CAR* chimeric antigen receptor

Patients meeting the eligibility criteria were enrolled and mononuclear cells were obtained by leukapheresis at the study site. The leukapheresed cells were shipped to the manufacturing site and processed to manufacture axi-cel, which was cryopreserved and shipped back to the study site. Bridging chemotherapy to control lymphoma between leukapheresis and conditioning chemotherapy was not permitted. Cyclophosphamide 500 mg/m^2^/day and fludarabine 30 mg/m^2^/day were administered for 3 consecutive days (− 5, − 4, and − 3) before axi-cel infusion as conditioning chemotherapy (also known as lymphodepleting chemotherapy; see supplementary materials for details). The number of cells was adjusted to the target dose of anti-CD19 CAR T cells (2.0 × 10^6^ cells/kg of body weight) (Fig. 1).

This study comprised stages 1 and 2 (Fig. S1). An interim analysis assessing ORR and safety was planned for early efficacy evaluation and early futility termination. The interim analysis was to be conducted with ten patients. If the drug was not deemed effective nor ineffective in the interim analysis, six patients were planned to be added. Since this is the first study in Japanese patients, the tolerability of axi-cel was also assessed once dose-limiting toxicity (DLT) evaluation period (28 days) for the first three subjects was finished (see supplementary materials).

The study protocol was approved by the independent ethics committees or institutional review boards of the study sites, and the study was conducted in accordance with the principles of the Declaration of Helsinki, International Conference on Harmonisation-Good Clinical Practice, and other applicable regulatory requirements. All patients provided written informed consent.

### Patients

Key inclusion criteria included the following: age ≥ 20 years; Eastern Cooperative Oncology Group performance status (ECOG PS) 0/1; histologically confirmed aggressive B-cell NHL (including DLBCL, Primary mediastinal large B-cell lymphoma (PMBCL), transformed follicular lymphoma (TFL), High-grade B-cell lymphoma (HGBCL) with *MYC* and *BCL2* and/or *BCL6* rearrangement; or HGBCL not otherwise specified) as defined by the World Health Organization (WHO) 2016 criteria [12]; chemorefractory disease or relapse ≤ 12 months after autologous stem cell transplantation (ASCT); prior use of an anti-CD20 monoclonal antibody and anthracycline-containing chemotherapy or prior chemotherapy for follicular lymphoma in patients with TFL; no evidence of central nervous system (CNS) lymphoma; absolute neutrophil count ≥ 1000/μL; platelet count ≥ 75,000/μL; and absolute lymphocyte count ≥ 100/μL.

Key exclusion criteria included a history of malignancy within the past 3 years, prior allogeneic stem cell transplantation, prior CD19-targeted therapy, prior CAR T-cell therapy or other genetically modified T-cell therapy.

### Endpoints

Disease response was evaluated per the International Working Group (IWG) 2007 criteria [13]. The primary efficacy endpoint was ORR (defined as the proportion of patients who achieved CR or PR) based on investigator assessment. Secondary endpoints included ORR based on central diagnostic imaging evaluation (see supplementary materials); best response observed among all disease assessments by an investigator (defined in the order of CR, PR, stable disease [SD], progressive disease [PD], and not evaluable); CR rate; duration of response (DOR); time to response (TTR); progression-free survival (PFS); and OS. Pharmacokinetic endpoints such as the concentration of axi-cel in blood and safety endpoints such as DLTs, treatment-emergent adverse events (TEAEs), serious adverse events (SAEs), and adverse events (AEs) of special interest were also evaluated. TEAEs were graded according to the National Cancer Institute Common Terminology Criteria for Adverse Events (CTCAE) version 4.03 [14]. Cytokine release syndrome (CRS) was graded according to the criteria of Lee et al. [15].

### Statistical analysis

Considering the ORR was 26% in SCHOLAR-1 [4] and 82% in ZUMA-1 [6], the threshold ORR was selected as 26%, and the expected ORR was set at 60%. The study design was decided with reference to the two-stage designs optimal under the alternative hypothesis suggested by Mander and Thompson [16], with a one-sided significance level of 5% and power of ≥ 80%. Analyses were performed using SAS^®^ version 9.4 (SAS Institute Inc., Cary, NC, USA) and Phoenix WinNonlin version 8.1 (Certara G.K., Princeton, NJ, USA). ORR with exact two-sided 90% confidence interval (CI) and two-sided 95% CI based on the Clopper–Pearson method were calculated. The safety analysis set included all patients who received axi-cel infusion. The pharmacokinetic (PK) analysis set included patients in the safety analysis set who had ≥ 1 available PK data. The modified intent-to-treat (mITT) analysis set included patients who received axi-cel infusion at a dose of ≥ 1.0 × 10^6^ CAR T cells/kg. The primary efficacy analysis set included patients in the mITT analysis set with primary endpoint data. The interim analysis set included the first ten treated patients from the primary efficacy analysis set.

## Results

### Patient disposition

**Fig. 2:**
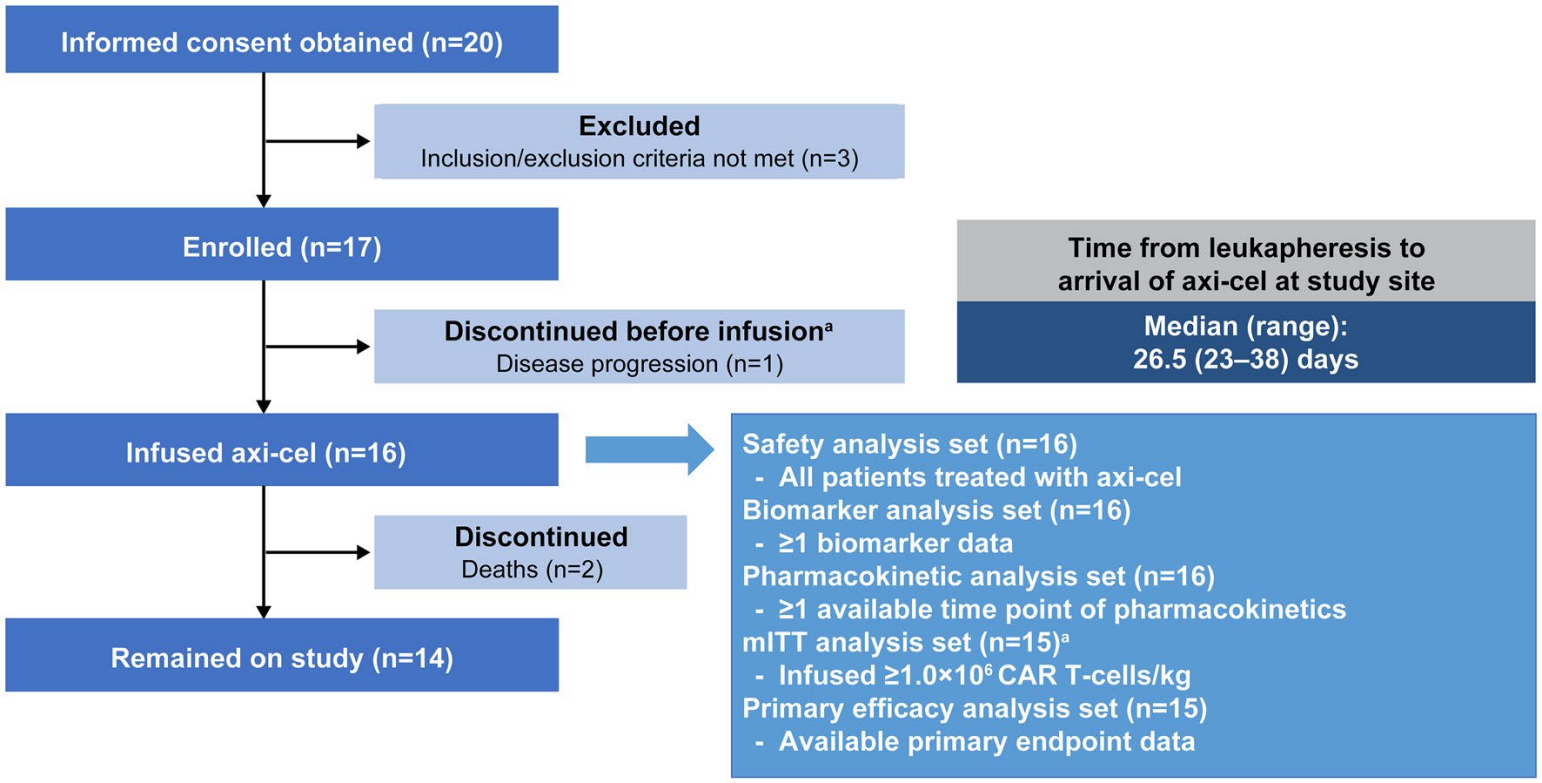
Patient disposition. ^a^Infusion was discontinued due to an anaphylactic reaction in one patient. This patient was excluded from the mITT analysis set because the infused axi-cel dose was < 1.0 × 10^6^ cells/kg of body weight. *axi-cel* axicabtagene ciloleucel, *CAR* chimeric antigen receptor, *mITT* modified intent-to-treat

The data cutoff date for the interim analysis was July 15, 2019 and that for the updated analysis was October 23, 2019. At data cutoff for the updated analysis, 20 patients had provided informed consent, of whom three patients did not meet the eligibility criteria and were excluded. Seventeen patients were enrolled in the study, and one patient underwent leukapheresis but discontinued the study before receiving conditioning chemotherapy due to disease progression. Sixteen patients received axi-cel and had the opportunity to be followed for a minimum of 3.0 months, with a median (range) actual follow-up time of 5.5 (3.0–10.4) months. In one patient, infusion was discontinued due to an anaphylactic reaction (considered as a reaction to dimethyl sulfoxide). This patient was included in the safety analysis set but excluded from the mITT analysis set due to an axi-cel infusion dose < 1.0 × 10^6^ cells/kg. Two patients died due to disease progression during the study and 14 patients remained on study (Fig. 2).

### Manufacturing and administration of axi-cel

The median (range) time from leukapheresis to the receipt of axi-cel at the study site was 26.5 (23–38) days (*n* = 16). The median (range) time from leukapheresis to axi-cel infusion was 29 (25–48) days. A total of 15 patients received the target dose of axi-cel (2.00 × 10^6^ cells/kg); one patient with the anaphylactic reaction received 0.4 × 10^6^ cells/kg of axi-cel. Axi-cel was manufactured in the United States and shipped and administered to patients in Japan.

### Patient demographics and baseline clinical characteristics

Of the 16 patients who received axi-cel, 14 (87.5%) were diagnosed with DLBCL per investigator assessment. The median (range) age was 58 (44–70) years, and five (31.3%) patients aged ≥ 65 years were included. Seven (43.8%) patients had stage IV disease. At baseline, 13 (81.3%) patients were CD19 positive and 1 patient was negative by immunohistochemistry (data were missing for 2 patients). Twelve (75.0%) patients had received ≥ 3 prior therapies, 10 (62.5%) patients were refractory to second- or subsequent-line therapy; and 6 (37.5%) patients relapsed after ASCT within a year (Table 1).

**Table 1:** Patient demographics and baseline clinical characteristics (safety analysis set, *n* = 16)

	Safety analysis set (*n* = 16)
Disease type by investigator, *n* (%)	
DLBCL	14 (87.5)^a^
PMBCL	1 (6.3)
TFL	1 (6.3)
HGBCL	0 (0.0)
Age	
Median (range), years	58 (44–70)
≥ 65 years, *n* (%)	5 (31.3)
Sex, *n* (%)	
Male	11 (68.8)
Female	5 (31.3)
Body weight, median (range), kg	63.6 (44.6–78.2)
ECOG PS, *n* (%)	
0	12 (75.0)
1	4 (25.0)
Disease stage at study entry^b^, *n* (%)	
I	4 (25.0)
II	4 (25.0)
III	1 (6.3)
IV	7 (43.8)
Bulky disease (≥ 1 lesion of 10 cm in diameter), *n* (%)	
Yes	1 (6.3)
No	15 (93.8)
Tumor burden (SPD) for target lesions, mm^2^	
Mean (SD)	4544.9 (6748.17)
Median (range)	1991.5 (288–26,360)
International Prognostic Index, *n* (%)	
0	3 (18.8)
1	4 (25.0)
2	3 (18.8)
3	4 (25.0)
4	2 (12.5)
5	0 (0.0)
CD19 positivity at baseline, *n* (%)	
Yes	13 (81.3)
No	1 (6.3)
Missing	2 (12.5)
≥ 3 lines of prior chemotherapy, *n* (%)	12 (75.0)
Refractory subgroup, *n* (%)	
Primary refractory	0 (0.0)
Refractory to second- or subsequent-line therapy	10 (62.5)
Relapse after ASCT	6 (37.5)

Baseline value is defined as the last value taken before conditioning chemotherapy

*ASCT* autologous stem cell transplantation, *CD* cluster of differentiation, *DLBCL* diffuse large B-cell lymphoma, *ECOG PS* Eastern Cooperative Oncology Group performance status, *HGBCL* high-grade B-cell lymphoma, *PMBCL* primary mediastinal large B-cell lymphoma, *SD* standard deviation, *SPD* sum of the products of the greatest diameters, *TFL* transformed follicular lymphoma

^a^One patient was confirmed as having HGBCL by central read assessment

^b^Cotswolds modification of the Ann Arbor staging system

### Efficacy

In the interim analysis, among the first 10 treated patients in the primary efficacy analysis set, CR or PR was observed in 9 patients and the prespecified criteria for efficacy was met. By the time the treatment was found effective in interim analysis, 17 patients had been already enrolled and 16 patients were treated consequently. In subsequent sections, data of the updated analysis for 16 patients with a minimum follow-up of 3.0 months (data cutoff: October 23, 2019) are described.

ORR based on investigator assessment in the primary efficacy analysis set was 86.7% (95% CI 59.5–98.3%; 13/15 patients) (Table 2). The best response was CR in 4 (26.7%) patients, PR in 9 (60.0%) patients, and SD and PD in 1 (6.7%) patient each (Table 2). The individual responses at each timepoint are shown in Table S1. The ORR by central imaging evaluation was 60.0% (95% CI 32.3–83.7%; 9/15 patients) with CR in four (26.7%) patients. Two patients were judged as not evaluable in central assessment. And the selected lesions for disease assessment were different from those selected in investigator assessment in some cases.

**Table 2:** ORR and best response based on investigator/assessment (primary efficacy analysis set; *n* = 15)

	*n* (%)	95% CI^a^
ORR^b^: CR + PR	13 (86.7)	59.5–98.3
Best response		
CR	4 (26.7)	7.8–55.1
PR	9 (60.0)	32.3–83.7
SD	1 (6.7)	0.2–31.9
PD	1 (6.7)	0.2–31.9
Not evaluable	0 (0.0)	0.0–21.8

*CI* confidence interval, *CR* complete response, *IWG* International Working Group, *ORR* objective response rate, *PD* progressive disease, *PR* partial response, *SD* stable disease

^a^Based on the Clopper–Pearson method

^b^Based on investigator assessment per the IWG 2007 criteria

**Fig. 3:**
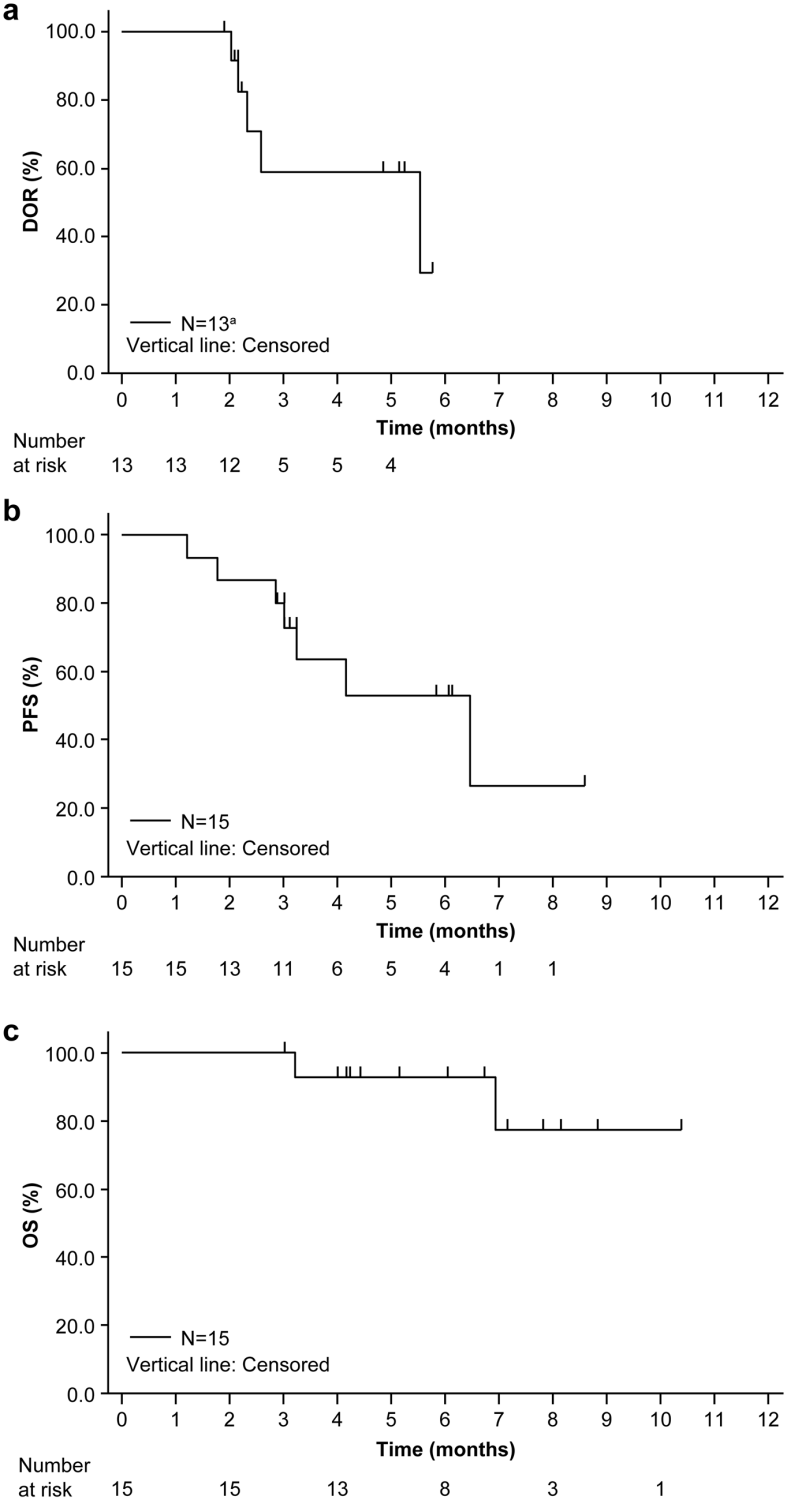
Kaplan–Meier estimates for (**a**) DOR (primary efficacy analysis set), (**b**) PFS (primary efficacy analysis set), and (**c**) OS (mITT analysis set). ^a^Patients who had responded to axi-cel in the primary efficacy analysis set. *axi-cel* axicabtagene ciloleucel, *DOR* duration of response, *mITT* modified intent-to-treat; *OS* overall survival, *PFS* progression-free survival

The median DOR (95% CI) was 5.6 (2.2–not estimable) months. Among 13 patients who responded to axi-cel, 8 (61.5%) showed an ongoing response (Fig. 3a). The median (range) TTR and time to CR were 0.95 (0.85–2.86) and 0.97 (0.95–1.05) months, respectively. Pseudo-progression was not observed. The median PFS (95% CI) was 6.5 (2.9–not estimable) months. Eight (53.3%) patients did not meet the criteria for disease progression or death (Fig. 3b). The median OS (95% CI) was not reached (6.9–not estimable), and 13 patients (86.7%) were alive at data cutoff (Fig. 3c).

### Safety

DLT was assessed with the first three enrolled patients and no DLT was observed. Grade ≥ 3 TEAEs occurred in 16 (100%) patients (Table S2). TEAEs related to conditioning chemotherapy and TEAEs related to axi-cel occurred in 16 (100%) patients each and were all grade ≥ 3. A TEAE (anaphylactic reaction) leading to discontinuation of axi-cel occurred in one (6.3%) patient with a history of infusion reaction to rituximab. Serious TEAEs occurred in 13 (81.3%) patients. No fatal TEAEs was reported. The most common TEAEs of any grade occurring in ≥ 8 patients were pyrexia (87.5%), lymphopenia (81.3%), neutropenia (81.3%), thrombocytopenia (75.0%), leukopenia (56.3%), decreased appetite (56.3%), diarrhea and nausea (50% each) (Table 3), while the most common grade ≥ 3 TEAEs that occurred in ≥ 8 patients were lymphopenia (81.3%), neutropenia (81.3%), thrombocytopenia (62.5%), and leukopenia (56.3%) (Table 3).

**Table 3:** TEAEs by CTCAE grade (safety analysis set; *n* = 16)

Preferred term^a^	All grade, *n* (%)	Grade ≥ 3, *n* (%)
Pyrexia	14 (87.5)	2 (12.5)
Lymphopenia^b^	13 (81.3)	13 (81.3)
Neutropenia^c^	13 (81.3)	13 (81.3)
Thrombocytopenia^d^	12 (75.0)	10 (62.5)
Leukopenia^e^	9 (56.3)	9 (56.3)
Decreased appetite	9 (56.3)	4 (25.0)
Diarrhea	8 (50.0)	3 (18.8)
Nausea	8 (50.0)	0 (0.0)
Febrile neutropenia	7 (43.8)	7 (43.8)
Anemia	7 (43.8)	5 (31.3)
Alanine aminotransferase increased	7 (43.8)	1 (6.3)
Aspartate aminotransferase increased	7 (43.8)	1 (6.3)
Malaise	6 (37.5)	0 (0.0)
Headache	5 (31.3)	0 (0.0)
Hypoxia	4 (25.0)	1 (6.3)
Hypophosphatemia	3 (18.8)	3 (18.8)
Hypogammaglobulinemia	3 (18.8)	2 (12.5)
Hyponatremia	3 (18.8)	1 (6.3)
Hypotension	3 (18.8)	1 (6.3)
Vomiting	3 (18.8)	0 (0.0)
Upper respiratory tract infection	3 (18.8)	0 (0.0)
Insomnia	3 (18.8)	0 (0.0)
Nasopharyngitis	3 (18.8)	0 (0.0)

CTCAE Version 4.03. TEAEs (all grade) that occurred in ≥ 3 patients are listed

*CTCAE* Common Terminology Criteria for Adverse Events, *MedDRA* Medical Dictionary for Regulatory Activities, *TEAE* treatment-emergent adverse event

^a^Coded per MedDRA version 21.0

^b^Lymphopenia includes lymphopenia and lymphocyte count decreased

^c^Neutropenia includes neutropenia and neutrophil count decreased

^d^Thrombocytopenia includes thrombocytopenia and platelet count decreased

^e^Leukopenia includes leukopenia and white blood cell count decreased

Treatment-emergent CRS was reported in 13 (81.3%) patients (12 cases of grade 1 or 2 and 1 case of grade 4). Eleven CRS cases were considered as SAEs. The most common symptoms of CRS were pyrexia (81.3%), diarrhea (18.8%), hypotension (12.5%), and hypoxia (12.5%) (Table S3). The median (range) duration from axi-cel infusion to first CRS event was 2.0 (1–11) days, and the median time to resolution was 16.5 days. Tocilizumab was administered in 11 (68.8%) patients. Steroids were administered in nine (56.3%) patients (for details of AE management, see Tables S4 and S5). No neurologic events or tumor lysis syndromes (TLSs) were reported. Among late-onset cytopenias (defined as any cytopenias present on or after 30 days), thrombocytopenia occurred in 12 (75.0%), neutropenia in 11 (68.8%), anemia in 5 (31.3%), and febrile neutropenia in 2 (12.5%) patients (Table 4). Among prolonged cytopenias (defined as any cytopenias with duration ≥ 30 days or consecutive events with a combined duration ≥ 30 days), neutropenia occurred in three (18.8%) patients; thrombocytopenia and anemia occurred in one (6.3%) patient each (Table 4). Three grade ≥ 3 infections were reported in two patients, with abdominal infection reported in one patient and acute sinusitis and infection reported in another patient (*n* = 1; 6.3% each); these events occurred more than 30 days after axi-cel infusion and resolved with medication and hospitalization or medication alone.

**Table 4:** Late-onset and prolonged cytopenias (safety analysis set; *n* = 16)

Adverse event Preferred term	Any grade, *n* (%)	Grade ≥ 3, *n* (%)
Late-onset cytopenias^a^		
Thrombocytopenia^b^	12 (75.0)	10 (62.5)
Neutropenia^c^	11 (68.8)	11 (68.8)
Anemia	5 (31.3)	4 (25.0)
Febrile neutropenia	2 (12.5)	2 (12.5)
Prolonged cytopenias^d^		
Neutropenia^c^	3 (18.8)	3 (18.8)
Thrombocytopenia^b^	1 (6.3)	0 (0.0)
Anemia	1 (6.3)	0 (0.0)

Coded with MedDRA version 21.0

*axi-cel* axicabtagene ciloleucel, *MedDRA* Medical Dictionary for Regulatory Activities

^a^Late-onset cytopenias were defined as any cytopenias present on or after 30 days of axi-cel infusion, including those that started after 30 days from axi-cel infusion and those that started earlier but were ongoing on or after 30 days of axi-cel infusion

^b^Thrombocytopenia includes thrombocytopenia and platelet count decreased

^c^Neutropenia includes neutropenia and neutrophil count decreased

^d^Prolonged cytopenias were defined as any cytopenias with duration ≥ 30 days or any consecutive events of cytopenias with a combined duration ≥ 30 days

The proportion of patients with detectable B cells in blood was 37.5% (6/16 patients) at baseline (prior to conditioning chemotherapy), 6.3% (1/16 patients) at week 4, 0% (0/15 patients) at month 3. Hypogammaglobulinemia occurred in three (18.8%) patients (grade ≥ 3: two [12.5%] patients), and all were treated with immunoglobulins.

No incidence or exacerbation of autoimmune disease or secondary malignancies was reported. All patients tested negative for antibody to FMC63 (the parental murine antibody used for development of the anti-CD19 single-chain variable fragment region of the CAR construct) and replication-competent retrovirus.

### CAR T cell expansion

**Fig. 4:**
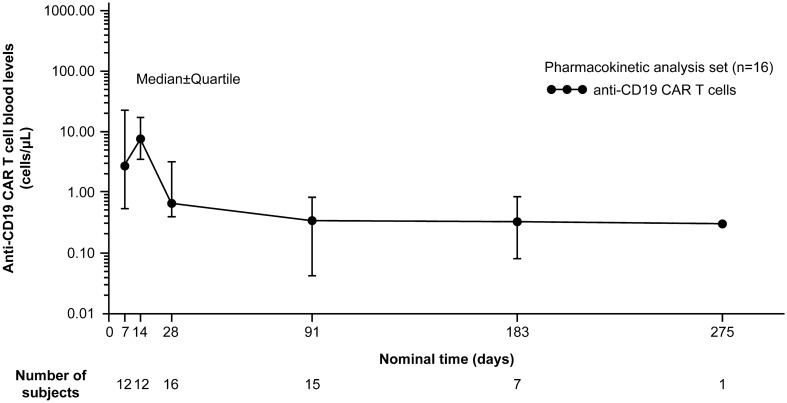
Median blood level of anti-CD19 CAR T cells. *CAR* chimeric antigen receptor, *CD* cluster of differentiation

Median anti-CD19 CAR T cell level in blood reached the maximum at 14 days after axi-cel infusion and decreased toward baseline (Fig. 4). The median (range) T_max_ for anti-CD19 CAR T cells in the blood, maximum blood concentration (C_max_), and area under the CAR T cells in blood-time curve up to 28 days (AUC_28d_) were 11 (7–28) days, 12.7 (0.9–297.4) cells/μL, and 187.5 (16.9–4244.5) cells × days/μL, respectively. At 6 months, CAR T cells were measurable in the blood for 6/7 evaluable patients. There was no clear correlation between CAR T cell expansion (AUC and peak level) and efficacy or severity of CRS in this study.

### Outcome of axi-cel re-administration

Axi-cel was re-administered in one patient whose clinical course before re-administration was CR at week 4 and month 3 and PD at month 6. CD19 expression on relapsed lymphoma tissue was confirmed by immunohistochemistry. Axi-cel manufactured for the first administration had been partially cryo-preserved as a second bag and it was re-administered on day 260. The pharmacokinetic parameters (T_max_, C_max_, and AUC_28d_) after re-administration were not significantly different from those for the first dose. The best response after re-administration was CR at week 4. The profile of TEAEs after re-administration was consistent with that reported for the main analysis.

## Discussion

This is the first study evaluating the efficacy, safety, and pharmacokinetics of axi-cel in Japanese patients. The primary endpoint ORR was 86.7% which is comparable with that in ZUMA-1 [6, 10], and higher than the historical data of SCHOLAR-1 (ORR 26%) [4]. Considering the patient baseline characteristics (e.g., 12 patients had received ≥ 3 prior lines of chemotherapy, and 6 patients relapsed within 12 months after ASCT), this ORR is encouraging. The CR rate in this study was lower than that in ZUMA-1 [6, 10], which can mainly be attributed to the shorter follow-up period. Interpretations of DOR and PFS are currently limited due to short observation period. Regarding CAR T cell expansion, median AUC_28d_ and median C_max_ levels were lower than those in ZUMA-1 but were within the range of variation in ZUMA-1 [6].

Finding biomarkers that can predict efficacy of axi-cel is important for patient stratification. Patients characteristic and/or product characteristics have been studied using ZUMA-1 data [17, 18]. It was suggested that low tumor burden, low systemic inflammation, and high product CCR7^+^CD45RA^+^ T cells were associated with durable responses [17].

No additional safety concerns were raised and no DLTs were observed in Japanese patients. The occurrence of common TEAEs, serious TEAEs, and AEs of special interest in this study was consistent with that in ZUMA-1 [6, 10]. CRS and immune effector cell-associated neurotoxicity syndrome are most frequently associated with CAR T-cell therapy and administration of tocilizumab, an anti-interleukin IL-6 receptor antibody, with or without steroids has been found to be effective in reversing CRS [19]. In this study, CRS occurred in 13 patients (1 patient of grade ≥ 3), and many of the events were managed with tocilizumab and steroid. No neurologic events occurred, the reason for which is however unknown. A real world data with large population will be necessary for further investigation.

Late-onset and prolonged cytopenias, other common adverse events related to CAR T-cell therapy [19], were observed in this study as well. A similar phenomenon was reported in a different study with a different product [20]. In the 24 months analysis of ZUMA-1, less than 20% of the patients had Grade ≥ 3 cytopenia at month 3 and beyond suggesting a gradual recovery of those cytopenias [10]. The proportion of patients with detectable B cells in blood was 37.5% at baseline, and 0% at month 3. In the ZUMA-1 study, B-cell recovery was observed in 75% of patients with an ongoing response at 24 months [10]. Future follow-up is necessary to ascertain the level of B-cell recovery in Japanese patients. Three grade ≥ 3 infections were reported more than 30 days after axi-cel infusion but all of them resolved with treatment.

Timely delivery is one of the key success factors for CAR T-cell therapy. Axi-cel arrived at study sites with the median time from leukapheresis to the receipt of 26.5 days. Among the 17 patients who underwent leukapheresis, 16 received axi-cel infusion. The low incidence of discontinuation can be attributed to the short turn-around time.

One patient who relapsed after CR was re-administered with axi-cel, and CR was achieved again. No significant additional safety concerns of re-administration were raised. To evaluate the clinical value of axi-cel re-administration, further investigation is required.

Since there is no head-to-head study, it is difficult to compare the efficacy and safety of different CAR T therapies. In a matching adjusted indirect comparison of axi-cel and tisa-cel, it was suggested that axi-cel may have superior efficacy but a greater risk of grade 1 or 2 CRS [21]. Future real world data will further clarify the difference of CAR T therapies.

In conclusion, axi-cel demonstrated clinically meaningful efficacy and a manageable safety profile in Japanese patients with R/R large B-cell lymphoma. The ORR and incidence of TEAEs observed in Japanese patients were comparable with those observed in the ZUMA-1 study and the bridging was feasible. The short turn-around time and low dropout rate in this study are promising factors for the use of axi-cel. Thus, axi-cel can be a good treatment option for Japanese patients with R/R large B-cell lymphoma. Limitations of this study include the small sample size and short follow-up period. Long-term follow-up is ongoing to determine conversions of response over time and identify any late-onset TEAEs. Analysis of mechanisms of disease progressions is also underway. Axi-cel is being evaluated for earlier line usage in large B-cell lymphoma, as well as in indolent NHL (NCT03391466, NCT03761056, NCT03105336) in the US and other parts of the world.

## Supplementary Information

Below is the link to the electronic supplementary material.

Supplementary file1 (DOCX 1242 KB)
