# Supplementary material for: Phase 2 study of axicabtagene ciloleucel in Japanese patients with relapsed or refractory large B-cell lymphoma
Source: Int J Clin Oncol. 2021 Oct 1;27(1):213–23. doi: 10.1007/s10147-021-02033-4 (PMC8732921; doi:10.1007/s10147-021-02033-4)
Supplement: Supplementary file 1 — Supplementary file1 (DOCX 1242 KB) [file 10147_2021_2033_MOESM1_ESM.docx]

**SUPPLEMENTARY MATERIALS**

**Article title:** Phase 2 study of axicabtagene ciloleucel in Japanese patients with relapsed or refractory large B-cell lymphoma

**Authors’ names**

Koji Kato, Shinichi Makita, Hideki Goto, Junya Kanda, Nobuharu Fujii, Kazuyuki Shimada, Koichi Akashi, Koji Izutsu, Takanori Teshima, Natsuko Fukuda, Tokuhito Sumitani, Hiroyuki Sumi, Shinji Shimizu, Yasuyuki Kakurai, Kenji Yoshikawa, Kensei Tobinai, Noriko Usui, Kiyohiko Hatake

**Corresponding author**

Koji Kato

Department of Medicine and Biosystemic Science

Kyushu University Graduate School of Medical Sciences

3-1-1, Maidashi, Fukuoka Higashi-ku

Fukuoka 812-8582, Japan

Telephone number: +81-92-642-5230

Fax number: +81-92-642-5247

Email address: [kato.koji.429@m.kyushu-u.ac.jp](mailto:kato.koji.429@m.kyushu-u.ac.jp)

**LIST OF SUPPLEMENTARY MATERIALS**

- **Schedule of Conditioning Chemotherapy**
- **Definition of Dose-Limiting Toxicities (DLT)**
- **Central Diagnostic Imaging Evaluation**
- **TABLE S1** Investigator evaluation at each time point
- **TABLE S2** Safety data (safety analysis set; n=16)
- **TABLE S3** Symptoms of CRS
- **TABLE S4** Management of CRS
- **TABLE S5** Management of neurologic events
- **Fig. S1** Flow of study
- **Schedule of Conditioning Chemotherapy**

Conditioning chemotherapy was administered for 3 days as described below :

- Intravenous (IV) hydration with 1 L of saline (0.9% sodium chloride [NaCl]) prior to cyclophosphamide on the day of infusion followed by:
- Cyclophosphamide 500 mg/m^2^ IV over 60 minutes on day −5, day −4, and day −3followed by:
- Fludarabine 30 mg/m^2^ IV over 30 minutes on day −5, day −4, and day −3 followed by:
- An additional 1 L of saline (0.9% NaCl) IV infusion at the completion of the fludarabine IV infusion
- Added mesna (sodium 2-mercaptoethanesulfonate) per institutional guidelines

**TABLE .** Schedule of conditioning chemotherapy

| **Day** | −**5** | −**4** | −**3** | −**2** | −**1** |
| --- | --- | --- | --- | --- | --- |
| Fludarabine  30 mg/m^2^/day | **X** | **X** | **X** | **-** | **-** |
| Cyclophosphamide 500 mg/m^2^/day | **X** | **X** | **X** | **-** | **-** |

- **Definition of Dose-Limiting Toxicities (DLT)**

DLTs were defined as KTE-C19-related AEs that occurred within 28 days after KTE-C19 administration and that met any of the following criteria.

- Grade 4 hematological AEs that persisted for longer than 30 days, not attributable to underlying conditions (excluding lymphocytopenia)
- All KTE-C19-related Grade 3 nonhematological AEs that persisted for more than 7 days, and all KTE-C19-related Grade 4 nonhematological AEs, except for the following:
  - Aphasia/dysphasia or confusion/cognitive impairment that recovered to at least Grade 1 or baseline within 2 weeks; or that recovered to at least baseline within 4 weeks
  - Grade 3 or Grade 4 fever
  - Anaphylaxis (KTE-C19-related) that occurred within 2 hours after KTE-C19 administration and that recovered to Grade 2 or below by standard treatment within 24 hours after KTE-C19 administration
  - Renal toxicity that required dialysis for ≤7 days
  - Events that required intubation for airway management for ≤7 days
  - TLS or signs associated with TLS (including events, such as electrolyte abnormality, impaired renal function, hyperuricemia)
  - Grade 3 liver laboratory abnormalities, such as transaminases, ALP, bilirubin, or other liver laboratory abnormalities, which recovered to ≤Grade 2 within 14 days
  - Transient Grade 4 liver laboratory abnormalities that recovered to ≤Grade 3 in less than 72 hours
  - Grade 3 or Grade 4 hypogammaglobulinemia
  - Grade 3 nausea or anorexia
- **Central Diagnostic Imaging Evaluation**

The central diagnostic imaging evaluation was conducted by Bioclinica, Inc. (211 Carnegie Center Drive Princeton, NJ 08540) as follows (data on file)

**Image Acquisition And Collection**

**1.0 Required On-Protocol Imaging**

Each patient will have an efficacy evaluation that includes a review of the radiographic studies performed while on-study. All images will be reviewed and response assessed using a modification of the International Harmonization Project/Cheson (2007) criteria

**1.1 Acceptable Imaging Modalities**

Image data that will be provided for the Independent Review will comprise whole body fluorodeoxyglucose positron emission tomography (FDG-PET) scans (skull base to proximal femurs, inclusive) and CT scans of the neck, chest, abdomen and pelvis (NCAP).

**1.2 Screening Images**

• PET-CT exams of the neck, chest, abdomen, and pelvis must be performed within 28 days prior to the start of conditioning chemotherapy and as close as possible to leukapheresis.

• PET-CT exams performed following the subject’s last line of therapy and prior to signing the informed consent may be used as the subject’s Screening exams, provided these exams were performed within 28 days prior to the start of conditioning chemotherapy.

• Additionally, PET-CT exams of other sites of primary or metastatic disease may be performed and submitted, if clinically indicated.

Note: for retreatment subjects, the new Screening time point will be the time point closest to (and prior to) the retreatment date that contains the required FDG-PET/CT NCAP imaging.

**1.3 On-Study Images (Assessment Period and Long-Term Follow-Up Period)**

On-Study imaging will be performed as indicated in the Schedule of Assessments in Section 1.5.

**1.4 Use of Contrast**

IV contrast is recommended when not medically contraindicated. If imaging of the abdomen and pelvis are required, the subject should be given oral contrast media, according to local imaging facility protocols.

**1.5 Schedule of Assessments**

The following table lists the required on-protocol imaging for this study.

**TABLE. Schedule of assessments**

| **Examination** | **Screening^a,b^** | **Assessment period^c,d^** | **Long-term follow-up^d,e^** |
| --- | --- | --- | --- |
| PET-CT neck | R | R | R |
| PET-CT chest | R | R | R |
| PET-CT abdomen | R | R | R |
| PET-CT pelvis | R | R | R |
| Additional imaging studies^f^ | A | A | A |

R: Required. Required examinations will be queried as “Missing” if not received.

A: Acceptable. If clinically indicated, additional examinations may be submitted on-study.

^a^Screening imaging must be performed within 28 days prior to the start of conditioning chemotherapy and as close as possible to leukapheresis.

^b^PET-CT exams performed following the patient’s last line of therapy and prior to signing the informed consent may be used as the patient’s screening exams, provided these exams were performed within 28 days prior to the start of conditioning chemotherapy.

^c^Assessment period imaging must be performed at week 4 (±3 days) and month 3 (±1 week).

^d^Patients with symptoms suggestive of disease progression should be evaluated by PET-CT at the time symptoms occur to verify the presence of disease progression.

^e^Long-term follow-up imaging must be performed at month 6 (±2 weeks), month 9 (±2 weeks), month 12 (±2 weeks), month 15 (±2 weeks), month 18 (± 2 weeks), and month 24 (±1 month), or until disease progression, whichever occurs first.

^f^Additional imaging studies may be performed and submitted at the discretion of the investigator.

- **TABLE S1** Investigator evaluation at each time point

| **Patient number** | **Month 1** | **Month 3** | **Month 6** | **Month 9** | **Note** |
| --- | --- | --- | --- | --- | --- |
| 1 | SD | PR | PR | PR |  |
| 2 | PR | PR | PR | PR |  |
| 3 | CR | CR | CR | - |  |
| 4 | CR | CR | CR | - |  |
| 5 | PR | PR | - | - |  |
| 6 | PR | PR | PR | - |  |
| 7 | CR | CR | CR | - |  |
| 8 | CR | CR | PD | - |  |
| 9 | PR | PR | PR | - |  |
| 10 | PR | PR | - | - | PD (unscheduled, day 127) |
| 11 | PR | PD | - | - |  |
| 12 | PR | PD | - | - |  |
| 13 | PR | PD | - | - |  |
| 14 | SD | - | - | - | PD (unscheduled, day 54) |
| 15 | PD | - | - | - |  |

CR, complete response; PD, progressive disease; PR, partial response; SD, stable disease.

- **TABLE S2** Safety data (safety analysis set; n=16)

|  | n (%) |
| --- | --- |
| **Any TEAE** | 16 (100.0) |
| Grade ≥3 | 16 (100.0) |
| **TEAEs related to conditioning chemotherapy** | 16 (100.0) |
| Grade ≥3 | 16 (100.0) |
| **TEAEs related to axi-cel** | 16 (100.0) |
| Grade ≥3 | 16 (100.0) |
| **Fatal TEAEs (excluding progressive disease)** | 0 (0.0) |
| **Serious TEAEs** | 13 (81.3) |
| **Treatment-emergent CRS** | 13 (81.3) |
| **Treatment-emergent neurologic events** | 0 (0.0) |
| **Treatment-emergent TLS** | 0 (0.0) |
| **TEAEs leading to interruption of axi-cel** | 1 (6.3) |

axi-cel, axicabtagene ciloleucel; CRS, cytokine release syndrome; TEAE, treatment-emergent adverse event; TLS, tumor lysis syndrome.

- **TABLE S3** Symptoms of CRS

| Preferred term^a^ | Any grade, n (%) | Grade ≥3,  n (%) |
| --- | --- | --- |
| Pyrexia | 13 (81.3) | 2 (12.5) |
| Diarrhea | 3 (18.8) | 2 (12.5) |
| Hypotension | 2 (12.5) | 1 (6.3) |
| Hypoxia | 2 (12.5) | 1 (6.3) |
| Alanine aminotransferase increased | 1 (6.3) | 1 (6.3) |
| Aspartate aminotransferase increased | 1 (6.3) | 1 (6.3) |
| Gamma-glutamyltransferase increased | 1 (6.3) | 1 (6.3) |
| Supraventricular tachycardia | 1 (6.3) | 1 (6.3) |
| Blood alkaline phosphatase increased | 1 (6.3) | 0 (0) |
| Blood pressure decreased | 1 (6.3) | 0 (0) |
| Fatigue | 1 (6.3) | 0 (0) |
| Headache | 1 (6.3) | 0 (0) |
| Malaise | 1 (6.3) | 0 (0) |
| Oxygen saturation decreased | 1 (6.3) | 0 (0) |
| Submaxillary gland enlargement | 1 (6.3) | 0 (0) |
| Vomiting | 1 (6.3) | 0 (0) |

^a^Severity grading per the CTCAE version 4.03.

CRS, cytokine release syndrome; CTCAE, Common Terminology Criteria for Adverse Events.

- **TABLE S4.** Management of CRS

**~~
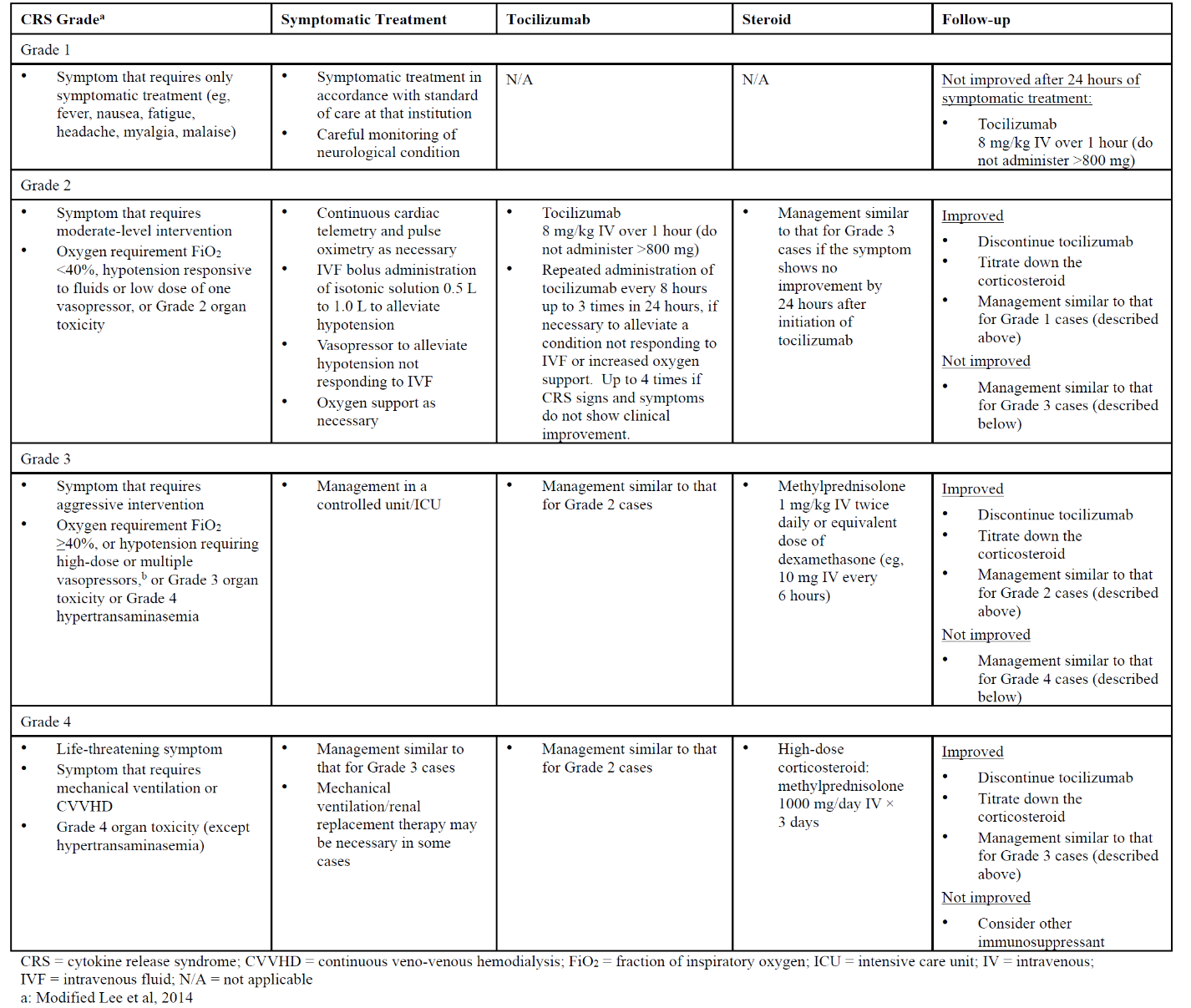
~~**

There were minor modifications in the management of CRS during the study period. The latest managements are shown.

- **TABLE S5.** Management of neurologic events


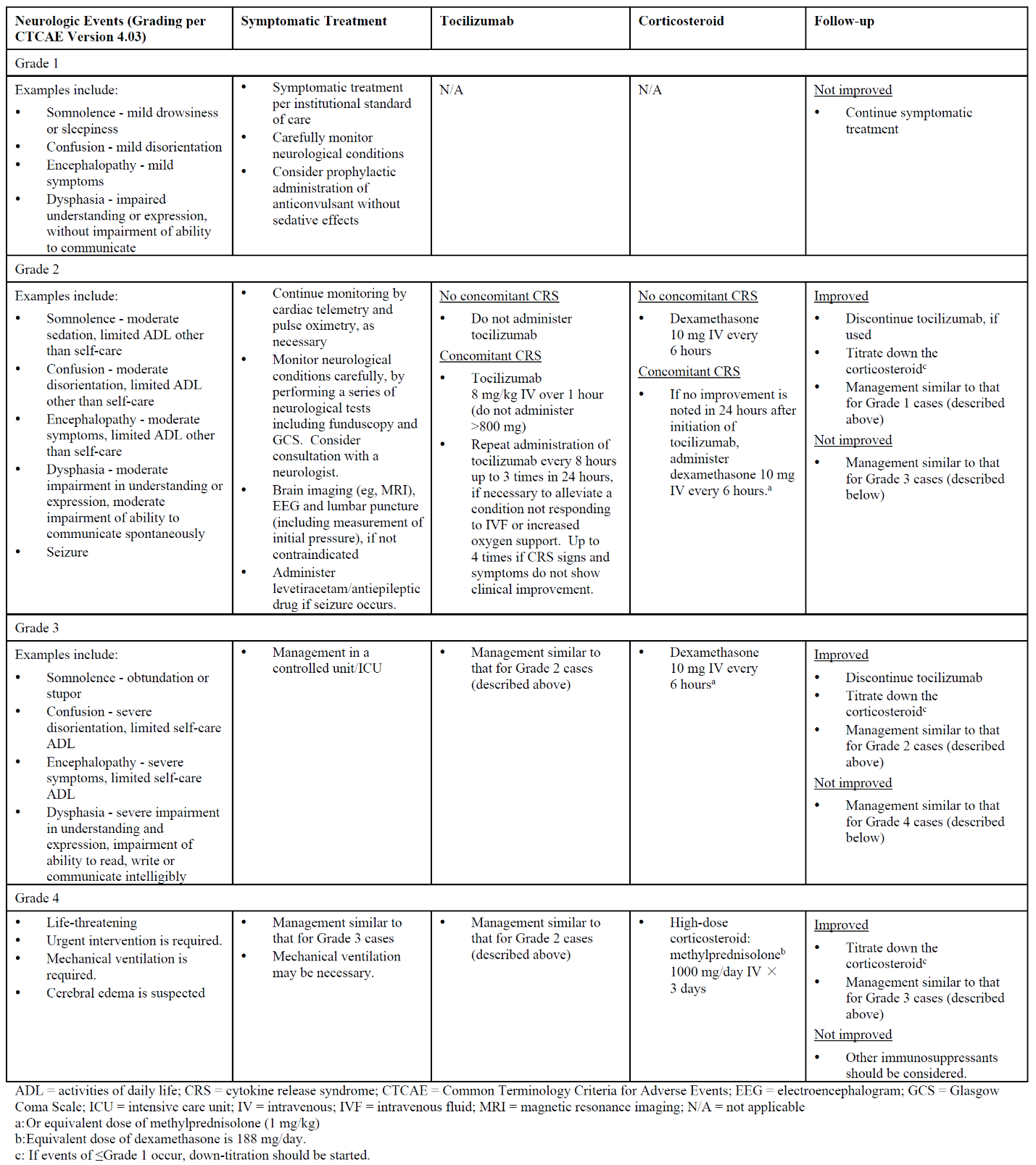


- **Supplementary Fig. 1** Flow of study


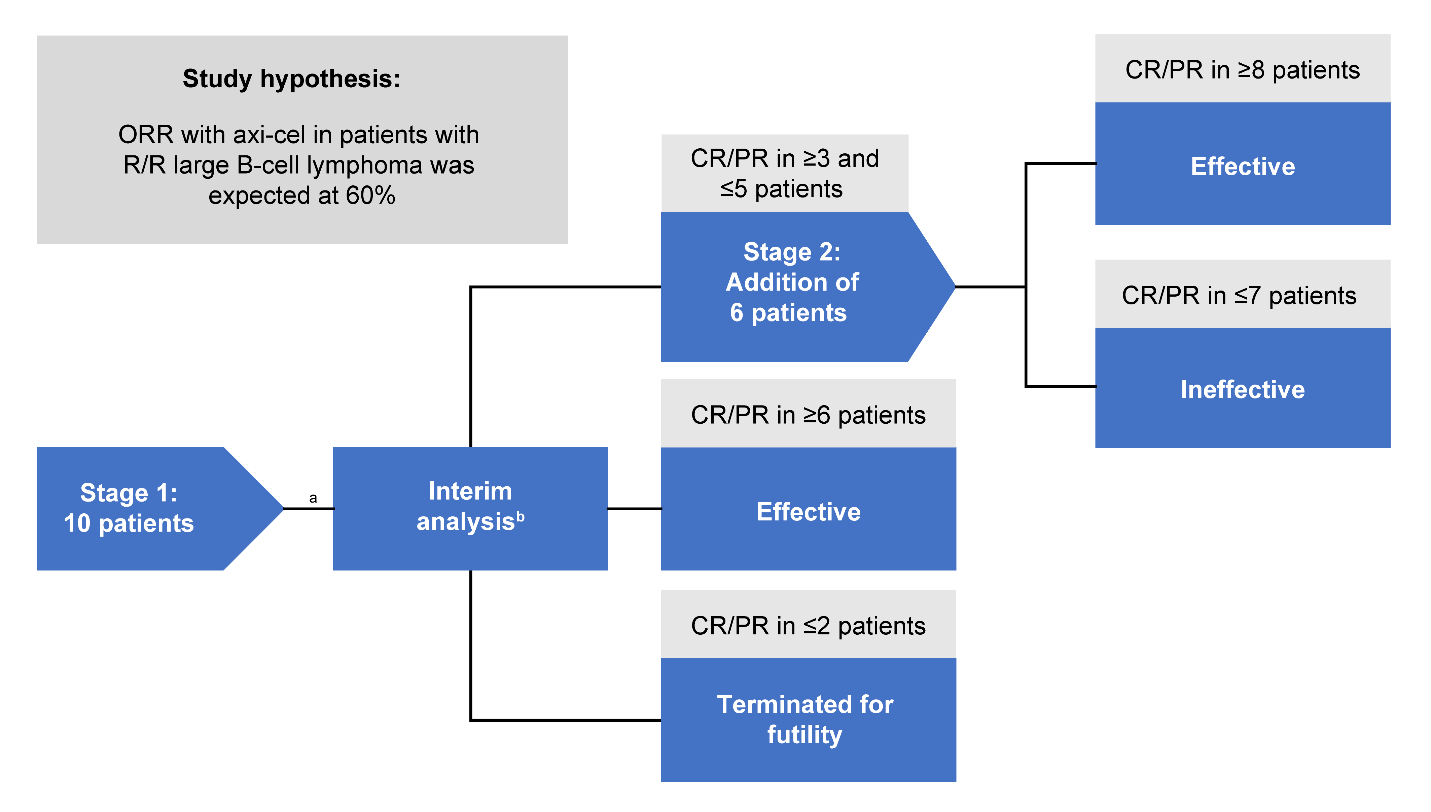


An interim analysis assessing ORR and safety was planned for early efficacy evaluation and early futility termination. The interim analysis was conducted when 10 patients in stage 1 had CR or PR, discontinued the study, or were followed up for 3 months during the study. Axi-cel was determined to be effective if CR or PR was achieved in ≥6 of 10 patients. If CR or PR was achieved in ≤2 of 10 patients, axi-cel was deemed to be ineffective and the study was to be terminated owing to futility at that point. If CR or PR was observed in 3–5 patients, the study was to be continued with the addition of six patients.

^a^Dose-limiting toxicity was assessed 28 days following axi-cel infusion after three patients were available.

**^b^**Patient enrollment was to be continued during the interim analysis.

axi-cel, axicabtagene ciloleucel; CR, complete response; ORR, objective response rate; PR, partial response; R/R, relapsed/refractory.
